# Exposure to Per- and Polyfluoroalkyl Substances and Risk of Psoriasis: A Population-Based Study

**DOI:** 10.3390/toxics12110828

**Published:** 2024-11-19

**Authors:** Qing Zhang, Mengyue Zhang, Cunxi Zhao

**Affiliations:** 1 School of Public Health, Anhui Medical University, Hefei 230032, China; zq18155498725@163.com; 2 Department of Clinical Medicine, The Second School of Clinical Medicine, Anhui Medical University, 81 Meishan Road, Hefei 230032, China; 2114010147@stu.ahmu.edu.cn

**Keywords:** per- and polyfluoroalkyl substances, psoriasis, mixture analysis, NHANES

## Abstract

PFAS are a group of synthetic chemicals that have been reported to be associated with adverse health outcomes. However, the relationship of PFAS exposure with psoriasis risk has not been reported. Utilizing data from the 2003–2018 NHANES, we explored the relationship of PFAS exposure with psoriasis risk. Our study included 5370 participants and examined serum levels of five PFAS compounds: PFOA, PFOS, PFHxS, PFNA, and PFDA, along with self-reported psoriasis status. Generalized linear regression, quantile g-computation, repeated hold out WQS regression, and BKMR models were employed to assess individual and combined effects of PFAS on psoriasis risk. We found each doubling the PFOS concentration was associated with a 19% increased risk of psoriasis (OR: 1.19; 95% CI: 1.01, 1.41) in the overall population. Sex-stratified analyses indicated significant associations between PFOA and PFNA exposure and psoriasis risk in females. Mixture analyses using WQS regression indicated that PFAS mixtures were associated with an 11% increased risk of psoriasis (OR: 1.11, 95% CI: 1.01, 1.22) in females in both the negative and positive direction. BKMR analyses also indicated a positive trend of PFAS mixtures with psoriasis risk in females. Our findings indicate a possible association between PFAS exposure and psoriasis risk, particularly in females.

## 1. Introduction

Per- and polyfluoroalkyl substances (PFAS) are a class of manmade chemicals that have been extensively utilized in a wide array of consumer products since the 1940s, owing to their remarkable hydrophobic, lipophobic, and heat-resistant properties [1,2,3]. These substances are exceptionally persistent in the environment and have been identified in human blood samples globally [4,5,6,7]. Human exposure to PFAS occurs primarily through ingestion and inhalation, including sources such as food supplies, drinking water, and dust [8].

Over the past few decades, a substantial body of research has investigated the adverse health effects linked to PFAS exposure. Human epidemiological studies have demonstrated strong associations of PFAS exposure in relation to various chronic diseases, such as cancers, adverse birth outcomes, immunotoxicity, and metabolic abnormalities [9,10,11,12,13,14,15]. Moreover, PFAS have been discovered to interact with a range of receptors and proteins, including fatty acid-binding proteins, peroxisome proliferator-activated receptors (PPARs), and estrogen receptors, resulting in their classification as endocrine-disrupting chemicals [16,17,18,19].

Psoriasis is a chronic inflammatory skin condition characterized by the excessive proliferation of keratinocytes and infiltration of immune cells, with adult prevalence estimated between 0.51% and 11.43%, and in children, it ranges from 0% to 1.37% [20]. The exact causes of psoriasis are still not fully elucidated, but it is widely proposed to be the result of a combination of genetic, immune system, and environmental factors [21,22,23,24]. Considering the immunomodulatory and endocrine-disrupting properties of PFAS [25,26,27], it is conceivable that exposure to these substances may contribute to the onset or exacerbation of psoriasis.

As of now, no study has explored the potential link between PFAS exposure and the risk of psoriasis. Consequently, this study was performed to fill that gap by comprehensively assessing the association of PFAS exposure with psoriasis using data from the NHANES, a representative sample of the US population. By employing a variety of statistical methodologies, including generalized linear regression, quantile g-computation, WQS regression, and BKMR models, we evaluate the individual and mixed effects of PFAS exposure on psoriasis risk. Furthermore, we perform subgroup analyses stratified by several socio-demographic factors and health-related behaviors to investigate potential differences in susceptibility to PFAS-associated psoriasis risk.

## 2. Materials and Methods

### 2.1. Study Design and Participants

In this secondary analysis study, we used publicly available data from the 2003–2018 NHANES, a program led by the NCHS (National Center for Health Statistics) at the CDC (Centers for Disease Control and Prevention). These survey cycles were selected as they included data on serum PFAS levels, self-reported psoriasis status, and participant demographic characteristics. The flowchart of study participant selection is presented in Supplemental Figure S1. The original NHANES program was designed and conducted by NCHS to assess the health and nutritional status of the civilian, non-institutionalized population in the US through a complex, multistage probability sampling method. All data collection procedures, including participant recruitment, surveys, and biological sample collection, were performed by NHANES staff. The NCHS Research Ethics Review Board granted approval for the comprehensive protocol of NHANES, ensuring all participants were included only after providing their written informed consent [28]. Our current analysis of this publicly available dataset was exempt from additional institutional review board approval.

### 2.2. Exposure Assessment

High-performance liquid chromatography-tandem mass spectrometry (HPLC-MS/MS) was used by the CDC to measure the serum concentrations of PFAS [29]. For concentrations below the limit of detection (LOD), values were assigned as the LOD/√2 [30]. PFAS with a detection frequency below 70% were excluded from the analysis. This study focused on five specific PFAS: PFOA, PFOS, PFHxS, PFNA, and PFDA. Due to the right-skewed distribution of PFAS concentrations, all PFAS were log_2_ transformed for statistical analyses.

### 2.3. Outcome Assessment

The data on psoriasis in this study were collected via self-reporting. Participants in the NHANES survey were asked a specific question in the health questionnaire: “Have you ever been told by a health care provider that you had psoriasis?” Those who answered “yes” to this question were classified as having a case of psoriasis for the purposes of this study [31].

### 2.4. Covariates

Several covariates known to be risk factors for psoriasis and factors influencing the serum PFAS levels were adjusted for based on previous studies [21,32] and using a directed acyclic graph (DAG) (Supplemental Figure S2). The included covariates were age (years, continuous); sex (male or female); race/ethnicity (Mexican-American, non-Hispanic White, non-Hispanic Black, other Hispanic, or other/multiracial); marital status (married or living with partner, unmarried, or other); educational attainment (lower than high school or greater than or equal to high school); poverty income ratio (PIR) (≤1.30, 1.31–3.50, or >3.50); smoking status (never, former, or current); alcohol consumption (never, former, mild, moderate, or heavy); body mass index (BMI) (kg/m^2^, continuous); physical activity (yes or no); healthy eating index (continuous); and urinary creatinine (μmol/L, continuous).

### 2.5. Statistical Analysis

In our research, we reported continuous variables using their mean and standard deviation (SD) or, alternatively, by their median and interquartile range (IQR). Categorical variables, on the other hand, were described through their frequency and respective proportions. When analyzing serum PFAS concentrations, we treated these as both categorical (tertiles) and continuous variables (log_2_ transformation). Participants were grouped based on their psoriasis status. To identify differences between these groups, we employed statistical tests such as Student’s *t*-tests and Mann–Whitney *U* tests for continuous variables and chi-square tests for categorical variables. We also computed Pearson correlation coefficients for log_2_ transformed PFAS levels.

For assessing the relationship of PFAS exposure with the risk of psoriasis, we used generalized linear regression models. Our analysis included an unadjusted model, followed by Model 1, which was adjusted for socio-demographic variables, and Model 2, which further included adjustments for health behavior characteristics, dietary data, and laboratory results. The relationship between PFAS levels and psoriasis was further scrutinized using restricted cubic spline (RCS) analysis, with set points at the 5th, 35th, 65th, and 95th percentiles of the PFAS levels after log_2_ transformation. To evaluate non-linear relationships, we used the ANOVA function in the R “rms” package, considering a *p*-value below 0.05 as indicative of non-linearity [33].

The mixed effects of five PFAS on psoriasis were examined using three approaches: quantile g-computation, repeated hold-out WQS regression, and the BKMR model. These approaches were instrumental in revealing both partial and overall dose–response relationships of environmental chemical mixtures with health outcomes, especially in the context of co-exposures that are highly correlated. All mixture models were adjusted for relevant covariates, and sex-specific associations were explored.

The first approach, quantile-based g-computation, allows individual mixture components to contribute positive or negative weights to a mixture index [34]. The “gqcomp” R package was used to estimate the overall change in psoriasis risk corresponding to a simultaneous one-quartile increase in all examined PFAS levels [34]. The estimates and 95% bootstrap confidence intervals were reported from 1000 iterations.

To assess the overall association between PFAS mixtures and psoriasis, we employed WQS regression as the second approach. This method can combine highly correlated exposures into a single weighted index while considering the relationships of each exposure with the outcome [35,36]. By introducing the weighted index into a regression model, the association of the index with psoriasis can be estimated. The weights, summing to 1, can be extracted to evaluate the importance of each mixture component to the outcome. To overcome the limitations of typical WQS regression, which divides the data into training and validation sets [37], we used a repeated holdout validation method, randomly partitioning the data 100 times to generate a distribution of validated results [38]. We calculated the WQS index for psoriasis using quartiles of PFAS concentrations (log_2_ transformed), with 40% of the data used for training and 60% for validation. The WQS index was calculated as the average of 100 bootstrap samples and incorporated into a multivariable logistic regression model, with β coefficients constrained in both negative and positive directions. The mean β coefficient from 100 repeated holdout validations was interpreted as the association of the PFAS mixture in relation to psoriasis. The final WQS index weights, calculated as the mean from the 100 holdouts, were interpreted as the relative importance of each individual PFAS in contributing to the overall association. PFAS biomarkers with weights greater than 1/c (c represents the number of components in the mixture) (0.20) were identified as significant chemicals of concern [35]. The analysis was performed using the “gWQS” package in R [36].

The third approach, BKMR, was employed to explore both linear and non-linear relationships, as well as potential interactions amongst various exposures [39]. This method involved a comprehensive process with 25,000 iterations, complemented by a convergence assessment through the Markov chain Monte Carlo technique. Univariate plots visualized the exposure–response association between each PFAS in the mixture, while the others were fixed at their median levels. The overall impact of the PFAS mixture was evaluated by fixing all PFAS levels at specific percentiles (ranging from the 25th to the 75th, in 5-percentile steps), with the median serving as a baseline for comparison. The investigation also included an analysis of the individual effect of each PFAS when other exposure biomarkers were set at the 25th, 50th, and 75th percentiles, specifically looking at the mean difference between the 75th and 25th percentiles. To ascertain the relative importance of each PFAS in relation to psoriasis, posterior inclusion probabilities (PIPs) were calculated, using a 0.5 threshold to highlight the most influential predictors [40]. All these procedures were conducted utilizing the “bkmr” package in R [41].

Stratified analyses using generalized linear regression models were performed to investigate potential effect modification by participant characteristics (age, sex, PIR, marital status, educational attainment, BMI, smoking status, alcohol consumption, and physical activity) on the PFAS–psoriasis association. Interaction terms between PFAS and each stratification variable were included to test for differences across subgroups, aiming to identify potential modifiers of the relationship of PFAS exposure with psoriasis risk and to better understand the complex interplay between PFAS, individual characteristics, and psoriasis.

All analyses were conducted using R software (version 4.1.2). The “survey” package was employed to account for the complex sampling design and obtain estimates representative of the US population [42]. A two-sided *p*-value < 0.05 was considered statistically significant.

## 3. Results

### 3.1. Baseline Characteristics of Study Participants

Table 1 describes the demographic profiles of the participants and compares the differences between the psoriasis and non-psoriasis groups. Of the 5370 individuals included in the study, 148 (2.76%) were identified with psoriasis. The average age across all participants was 46.50 years, and the gender distribution was nearly equal, with females comprising 50.40%. Notable disparities were found between the psoriasis and non-psoriasis groups in aspects like BMI, race/ethnicity, urinary creatinine levels, and smoking status. The study included five PFAS for analysis, and their concentrations are shown in Supplemental Table S1.

### 3.2. Associations Between Individual PFAS and Psoriasis Risk

Table 2 presents the effect estimates on the associations of individual PFAS exposures with risk of psoriasis. In the unadjusted models, both PFOA and PFOS showed a significant association with an elevated psoriasis risk for each doubling of exposure concentration. However, when adjusted for various covariates, only the relationship between PFOS exposure and psoriasis remained statistically significant (OR: 1.19; 95% CI: 1.01, 1.41). Evaluating PFAS exposures as categorical variables, the highest tertile for PFOA, PFOS, and PFHxS was linked with an increased psoriasis risk compared to the lowest tertile in the unadjusted models. After controlling for covariates, only the association between the third tertile of PFOS and psoriasis risk remained significant (OR: 1.74; 95% CI: 1.00, 3.02).

The subgroup analysis results based on participant characteristics are presented in Supplemental Tables S2–S6. For continuous PFAS exposure, significant associations were observed in the following subgroups: age ≥ 60 years, females, PIR ≥ 1.85, educational attainment ≥ high school, BMI ≤ 24.9, and never smokers for PFOA exposure; PIR ≥ 1.85, married or living with a partner, and former alcohol consumers for PFOS exposure; BMI ≤ 24.9 and former alcohol consumers for PFHxS exposure; current smokers for PFDA exposure; and females for PFNA exposure. The categorical PFAS exposure analysis yielded similar results for each individual PFAS.

Considering the sex-specific associations of PFAS exposure with psoriasis risk, we performed sex-stratified multivariable-adjusted RCS analyses for the five PFAS to explore the dose–response relationships. A non-linear association was observed between PFOA and psoriasis in females (*p* for overall association < 0.05 and *p* for non-linearity < 0.05, Supplemental Figure S3B). However, no significant linear or non-linear associations were found for the other PFAS in either males or females (Supplemental Figures S3–S7).

### 3.3. Associations Between PFAS Mixture and Psoriasis Risk

#### 3.3.1. Sex-Specific Analysis of PFAS Mixture and Psoriasis Using Quantile G-Computation

Quantile g-computation was performed on the overall population and stratified by sex. The analyses revealed no significant associations between increased serum concentrations of the five PFAS per quantile and psoriasis risk in the overall population (OR: 1.11, 95% CI: 0.90, 1.37), males (OR: 1.04, 95% CI: 0.79, 1.37), or females (OR: 1.22, 95% CI: 0.89, 1.67). The detailed weights for each PFAS in the subset analyses are presented in Supplemental Figure S8. Notably, PFDA was assigned the largest negative weight of 0.893 in the overall population analysis, while PFNA and PFOA were assigned the largest positive weights of 0.503 and 0.772 in the male and female subgroup analyses, respectively.

#### 3.3.2. Sex-Specific Analysis of PFAS Mixture and Psoriasis Using WQS

**Figure 1:**
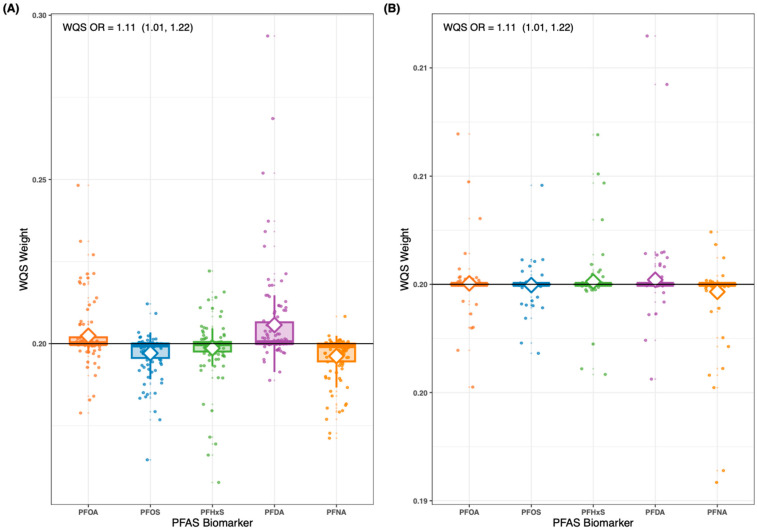
Empirical weights estimated from repeated holdout validation of PFAS biomarkers indicating relative contribution to the PFAS index for (**A**) the positive direction model or (**B**) negative direction model. Boxplots show the 25th, 50th, and 75th percentiles with the whiskers as 1.5 times the interquartile range (IQR) for 100 holdouts. White diamonds show mean weights for the 100 holdouts. Black line indicates the concern threshold (threshold = 0.20). The association with the WQS index is embedded as text.

Repeated holdout WQS regression was performed on the overall population and stratified by sex, considering both negative and positive directions. In the positive direction, the PFAS mixture was not related to psoriasis in the overall population (OR: 1.04, 95% CI: 0.79, 1.37) or in males (OR: 1.00, 95% CI: 0.91, 1.11) but was associated with an increased risk in females (OR: 1.11, 95% CI: 1.01, 1.22) (Figure 1A). Conversely, in the negative direction, the PFAS mixture was related to psoriasis in the overall population (OR: 1.06, 95% CI: 1.00, 1.13) and in females (OR: 1.11, 95% CI: 1.01, 1.22) (Figure 1B) but not in males (OR: 1.00, 95% CI: 0.91, 1.10). In the positive direction model for females, PFDA was assigned the highest individual weight within the index (mean weight = 0.21) (Figure 1A). In contrast, the negative direction model for females assigned equal weights of 0.20 to each PFAS (Figure 1B).

#### 3.3.3. Sex-Specific Analysis of PFAS Mixture and Psoriasis Using BKMR

**Figure 2:**
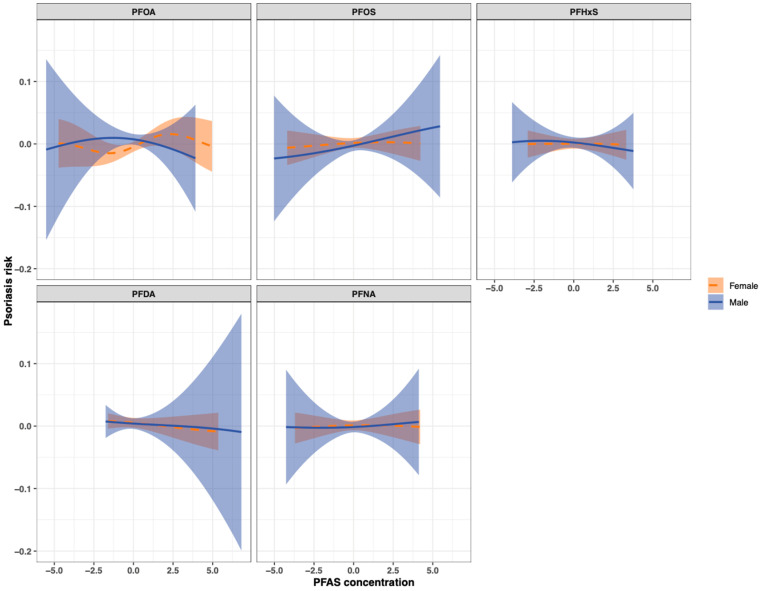
Sex-specific BKMR univariate exposure–response plots with the 95% credible intervals for the effect of each biomarker when others are fixed at their median.

**Figure 3:**
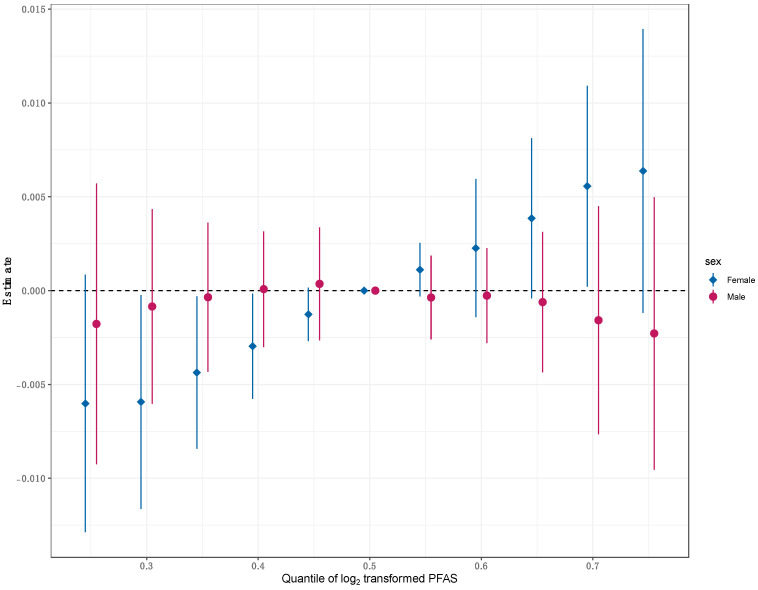
Sex-specific joint effects of the overall PFAS mixture on psoriasis where graphs depict the effect estimated (with a 95% credible interval) when all phthalate biomarkers are fixed at a particular percentile (x-axis) compared to when all biomarkers are at their respective median (reference).

**Figure 4:**
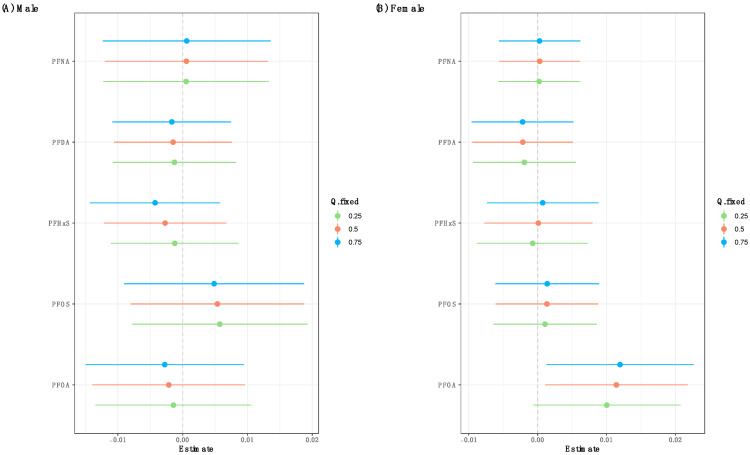
Sex-specific associations of each individual PFAS with psoriasis risk in the BKMR model. This plot describes the estimated psoriasis risk associated with a change in each individual PFAS from its 25th to 75th percentile, when all the other PFAS are fixed at the 25th, 50th, or 75th percentile. Dots indicate the estimate, and horizontal lines indicate the 95% credible intervals.

The BKMR univariate exposure–response analysis on the overall population revealed non-linear associations between the five PFAS and psoriasis (Supplemental Figure S9). Upon evaluating the cumulative impact of the mixture, neither a discernible positive or negative trend was evident (Supplemental Figure S10). The single-exposure association analysis suggested that PFOA exposure was associated with lower odds of psoriasis when all other exposure biomarkers were fixed at the 50th percentile (Supplemental Figure S11). Sex-stratified BKMR analysis revealed non-linear associations between PFOA and psoriasis in both males and females, while PFOS was associated with a modest increase in psoriasis risk in males (Figure 2). Stratifying the analysis by sex revealed a positive trend in the overall mixture effect among females, implying that elevating all exposures in the mixture was linked to a non-significant increase in psoriasis risk (Figure 3). When conducting sex-specific single-exposure association analyses, we found that PFOA exposure was related to increased odds of psoriasis when fixing all other exposure biomarkers at the 25th and 50th percentiles (Figure 4). The BKMR analysis did not identify any individual PFAS as an “important” predictor of psoriasis in the overall population or in the sex-stratified subgroups, as evident from the PIPs < 0.5 for all PFAS.

## 4. Discussion

This nationally representative study used data from NHANES to investigate the association of PFAS exposure with psoriasis risk. Our findings suggest that exposure to certain individual PFAS, especially PFOS, was related to an elevated risk of psoriasis in the general population. Sex-stratified analyses revealed significant associations of PFOA and PFNA exposure with psoriasis risk among females. Furthermore, mixture analyses indicated that exposure to PFAS mixtures was associated with an increased risk of psoriasis in females. To our knowledge, this study represents the first comprehensive examination of the relationship between PFAS exposure and psoriasis risk in a representative US population.

Our results align with previous studies that have connected PFAS exposure to various immune-related disorders. For instance, a systematic review and meta-analysis by Rappazzo et al. reported an association of PFAS exposure with an increased risk of asthma and allergic diseases in children and adolescents [43]. Similarly, a study by Qu et al. discovered that higher serum levels of PFOA were associated with an elevated risk of rheumatoid arthritis in a Chinese population [44]. These studies support the hypothesis that PFAS may disrupt immune function and contribute to the development of autoimmune diseases, including psoriasis.

The observed gender-specific association of PFAS exposure with psoriasis risk in our study is intriguing. Previous research has shown that women are more susceptible to autoimmune diseases, including psoriasis, compared to men [45]. This difference may be attributed to the complex interplay between hormonal factors, genetic susceptibility, and environmental exposures [46,47]. Relevant studies indicate that PFAS possess properties that can disrupt endocrine function, potentially affecting estrogen signal pathways [48,49,50] and was reported to be associated with androgen indicators in postmenopausal women [51]. As estrogen plays a crucial role in modulating immune responses [52,53,54,55], it is plausible that PFAS-induced hormonal imbalances may contribute to the increased risk of psoriasis in women.

While the precise mechanisms linking PFAS exposure to psoriasis risk remain to be elucidated, several potential pathways have been suggested. Studies have demonstrated that PFAS can induce oxidative stress and inflammation [56,57,58], which are critical factors in the pathogenesis of psoriasis [59,60,61]. Moreover, PFAS may influence the gut microbiome [62,63], which has been implicated in the development of psoriasis [64,65,66,67]. Additionally, PFAS have been shown to modulate the function of immune cells, such as T cells and macrophages [68,69,70], which are key players in the pathogenesis of psoriasis [71].

Our findings highlight the importance of assessing the impact of PFAS mixtures on the risk of psoriasis. In real-life scenarios, individuals are often exposed to multiple PFAS compounds rather than a single PFAS. There may be complex interactions among different PFAS, leading to synergistic or antagonistic effects. Therefore, evaluating the association between a single PFAS and psoriasis risk may underestimate the overall impact of PFAS mixtures. We employed various statistical methods, such as quantile g-computation, WQS regression, and BKMR models, to more comprehensively assess the relationship between PFAS mixtures and psoriasis risk. This integrated analytical approach provides a new perspective on understanding the health effects of environmental pollutant mixtures [72,73].

The findings of our research hold considerable significance for the formulation of public health strategies. Given the potential association between PFAS exposure and increased risk of psoriasis, it is necessary to develop strategies to reduce PFAS exposure levels in the population. This may include stricter regulations on PFAS production and use, the development of safer alternatives, and improved detection and purification technologies for PFAS in drinking water and food [74,75,76]. Additionally, for individuals at a high risk of psoriasis, such as women with higher levels of PFAS exposure, more frequent screening and monitoring may be needed for early detection and intervention of psoriasis.

This study has several strengths. We used data from a sizable, nationally representative sample, thereby increasing the generalizability of our findings. We also employed multiple statistical approaches, including generalized linear regression, quantile g-computation, WQS regression, and BKMR models, to assess the individual and combined effects of PFAS exposure on psoriasis risk. This comprehensive analysis provides a more robust understanding of the complex relationship between PFAS mixtures and psoriasis. Additionally, by stratifying our analysis by sex, we were able to identify sex-specific associations of PFAS exposure with psoriasis risk. However, it is important to highlight several limitations of our study. Firstly, the cross-sectional nature of the NHANES data prevents us from establishing a causal relationship between PFAS exposure and psoriasis risk. Secondly, the reliance on self-reported information for the diagnosis of psoriasis may introduce recall bias. Third, the lack of information on the severity or subtype of psoriasis may have obscured potential differences in associations with PFAS exposure. Lastly, although we adjusted for some potential confounding factors, the possibility of residual confounding by unmeasured factors cannot be entirely eliminated. To address these identified limitations, several methodological strategies could be implemented in future research. First, the establishment of large-scale prospective cohort studies with repeated PFAS measurements and standardized psoriasis assessments would help overcome both the cross-sectional design limitation and recall bias from self-reporting. Second, the implementation of a dual validation approach, combining both clinical records and patient self-reports, along with photographic documentation of lesions, could significantly reduce the misclassification bias in psoriasis diagnosis. Third, the adoption of standardized clinical assessment tools (such as PASI scores) and detailed subtype classification protocols would provide more comprehensive disease characterization. Finally, to minimize residual confounding, future studies should employ advanced statistical methods such as propensity score matching, negative control analyses, or E-value calculations to quantify the potential impact of unmeasured confounders. Additionally, the collection of more detailed covariate information, particularly regarding genetic factors and environmental co-exposures, would help reduce residual confounding.

## 5. Conclusions

In summary, our study offers the first evidence pointing to a potential relationship of PFAS exposure with an elevated risk of psoriasis, with a more pronounced effect among women. These findings highlight the necessity of assessing the potential health impacts of PFAS exposure, not only on well-established metabolic and cardiovascular diseases but also on immune-related disorders such as psoriasis. To substantiate our findings and unravel the underlying biological pathways, additional research, including prospective cohort studies and mechanistic explorations, is imperative. Moreover, our results provide new insights that can guide the development of public health interventions aimed at mitigating PFAS exposure and its potential deleterious health consequences.

## Figures and Tables

**Table 1:** Baseline characteristics of participants by psoriasis status, NHANES 2003–2018 (N = 5370).

Characteristics	Overall (N = 5370)	Non-Psoriasis (N = 5222)	Psoriasis (N = 148)	*p*-Value
Age, years (mean ± SD)	46.50 ± 16.83	46.43 ± 16.86	49.14 ± 15.79	0.05
BMI, kg/m^2^ (mean ± SD)	29.13 ± 6.98	29.05 ± 6.94	32.01 ± 8.05	<0.001
PIR, (mean ± SD)	2.61 ± 1.64	2.60 ± 1.64	2.73 ± 1.61	0.36
HEI, (mean ± SD)	50.53 ± 13.55	50.57 ± 13.59	49.11 ± 11.96	0.20
Urinary creatinine, μmol/L (mean ± SD)	11,285.82 ± 7166.17	11,324.60 ± 7187.50	9913.96 ± 6547.51	0.02
Sex, n (%)				0.50
Male	2666 (49.6)	2588 (49.6)	78 (52.7)	
Female	2704 (50.4)	2634 (50.4)	70 (47.3)	
Race/Ethnicity, n (%)				< 0.001
Non-Hispanic White	2580 (48.0)	2487 (47.6)	93 (62.8)	
Non-Hispanic Black	1111 (20.7)	1088 (20.8)	23 (15.5)	
Mexican American	808 (15.0)	800 (15.3)	8 (5.4)	
Other	871 (16.2)	847 (16.2)	24 (16.2)	
Marital status, n (%)				
Unmarried or other	2098 (39.1)	2047 (39.2)	51 (34.5)	0.28
Married or living with a partner	3272 (60.9)	3175 (60.8)	97 (65.5)	
Educational attainment, n (%)				0.84
<High school	1217 (22.7)	1185 (22.7)	32 (21.6)	
≥High school	4153 (77.3)	4037 (77.3)	116 (78.4)	
Alcohol consumption, n (%)				0.23
Never	698 (13.0)	684 (13.1)	14 (9.5)	
Former	942 (17.5)	909 (17.4)	33 (22.3)	
Mild	1750 (32.6)	1702 (32.6)	48 (32.4)	
Moderate	851 (15.8)	823 (15.8)	28 (18.9)	
Heavy	1129 (21.0)	1104 (21.1)	25 (16.9)	
Smoking status, n (%)				0.001
Never	2977 (55.4)	2910 (55.7)	67 (45.3)	
Former	1230 (22.9)	1178 (22.6)	52 (35.1)	
Current	1163 (21.7)	1134 (21.7)	29 (19.6)	
Physical activity, n (%)				0.37
No	2436 (45.4)	2363 (45.3)	73 (49.3)	
Yes	2934 (54.6)	2859 (54.7)	75 (50.7)	

Abbreviations: SD, standard deviation; BMI, body mass index; PIR, poverty income ratio; HEI, healthy eating index. The HEI is composed of food groups and nutrient indicators that score for adequacy or moderation. PIR: calculated by dividing the family income by the poverty threshold. Alcohol consumption: “Never” <12 drinks in lifetime, “Former” ≥12 drinks in 1 year and no drink last year or no drink last year but ≥12 drinks in lifetime, “Mild” <1 drinks/d for female and <2 drinks/d for male, “Moderate” 1 to 2 drinks/d is for females and 2 to 3 drinks/d is for males, and “Heavy” ≥3 drinks/d is for females and ≥4 drinks/d is for males. Physical activity: “Yes” if engaging in moderate-intensity or vigorous-intensity sports, fitness, or recreational activities for > 10 min on a typical day; otherwise, “No”.

**Table 2:** Associations between single serum per- and polyfluoroalkyl substance (PFAS) and odds of psoriasis in the NHANES 2003–2018 cycles.

PFAS (ng/mL)	Unadjusted		Model 1		Model 2	
	OR (95% CI)	*p-*Value	OR (95% CI)	*p-*Value	OR (95% CI)	*p-*Value
**PFOA**						
Per doubling of exposure	1.26 (1.03, 1.54)	**0.03**	1.19 (0.95, 1.50)	0.13	1.22 (0.97, 1.52)	0.09
T1	Ref	-	Ref	-	Ref	-
T2	1.40 (0.79, 2.46)	0.20	1.30 (0.73, 2.32)	0.40	1.44 (0.80, 2.58)	0.20
T3	1.99 (1.08, 3.65)	**0.03**	1.78 (0.90, 3.51)	0.10	1.91 (0.98, 3.72)	0.06
**PFOS**						
Per doubling of exposure	1.20 (1.04, 1.39)	**0.02**	1.16 (0.99, 1.37)	0.07	1.19 (1.01, 1.41)	**0.04**
T1	Ref	-	Ref	-	Ref	-
T2	1.41 (0.76, 2.59)	0.30	1.30 (0.70, 2.42)	0.40	1.35 (0.72, 2.56)	0.30
T3	1.78 (1.06, 2.97)	**0.03**	1.60 (0.92, 2.77)	0.10	1.74 (1.00, 3.02)	**0.05**
**PFHxS**						
Per doubling of exposure	1.15 (0.97, 1.36)	0.11	1.09 (0.91, 1.31)	0.30	1.11 (0.92, 1.33)	0.30
T1	Ref	-	Ref	-	Ref	-
T2	1.37 (0.87, 2.18)	0.20	1.24 (0.79, 1.97)	0.30	1.32 (0.84, 2.07)	0.20
T3	1.73 (1.09, 2.77)	**0.02**	1.50 (0.91, 2.49)	0.11	1.64 (0.98, 2.75)	0.06
**PFDA**						
Per doubling of exposure	1.00 (0.85, 1.18)	0.99	0.99 (0.83, 1.19)	0.93	1.03 (0.85, 1.23)	0.80
T1	Ref	-	Ref	-	Ref	-
T2	0.49 (0.30, 0.81)	**0.01**	0.48 (0.29, 0.80)	**0.01**	0.49 (0.30, 0.82)	**0.01**
T3	0.84 (0.54, 1.31)	0.40	0.80 (0.50, 1.30)	0.40	0.85 (0.51, 1.41)	0.50
**PFNA**						
Per doubling of exposure	1.14 (0.97, 1.35)	0.12	1.11 (0.92, 1.33)	0.30	1.13 (0.95, 1.35)	0.20
T1	Ref	-	Ref	-	Ref	-
T2	0.84 (0.49, 1.43)	0.50	0.80 (0.47, 1.37)	0.40	0.88 (0.52, 1.48)	0.60
T3	1.35 (0.87, 2.10)	0.20	1.25 (0.76, 2.06)	0.40	1.32 (0.80, 2.18)	0.30

Abbreviations: OR, odds ratio; CI, confidence interval; T, tertile; PFOA, perfluorooctanoic acid; PFOS, perfluorooctane sulfonate; PFHxS, perfluorohexane sulfonic acid; PFDA, perfluorodecanoic acid; PFNA, perfluorononanoic acid. PFAS concentrations were log_2_ transformed. Model 1 was adjusted for age, sex, race/ethnicity, educational attainment, marital status, and PIR. Model 2 was adjusted for age, sex, race/ethnicity, educational attainment, marital status, PIR, smoking status, alcohol consumption, healthy eating index, physical activity, and urinary creatinine. Bold values indicate statistical significance (*p* < 0.05).

## Data Availability

The datasets used for these analyses are publicly available (https://www.cdc.gov/nchs/nhanes/index.htm, accessed on 13 November 2024). The code for the statistical analysis will be provided as required. The raw data supporting the conclusions of this article will be made available by the authors on request.

## Supplementary Materials

The following supporting information can be downloaded at https://www.mdpi.com/article/10.3390/toxics12110828/s1: Figure S1: Flowchart of participants selection from the NHANES 2003–2018; Figure S2: Directed acyclic graph (DAG) for covariate selection; Figure S3: The restricted cubic spline analysis of dose–response relationships between PFOA and risk of psoriasis for (A) males and (B) females; Figure S4: The restricted cubic spline analysis of dose–response relationships between PFOS and risk of psoriasis for (A) males and (B) females; Figure S5: The restricted cubic spline analysis of dose–response relationships between PFHxS and risk of psoriasis for (A) males and (B) females; Figure S6: The restricted cubic spline analysis of dose–response relationships between PFDA and risk of psoriasis for (A) males and (B) females; Figure S7: The restricted cubic spline analysis of dose–response relationships between PFNA and risk of psoriasis for (A) males and (B) females; Figure S8: Positive and negative weights representing the partial effects of individual PFAS in the mixture on psoriasis risk, estimated using quantile g-computation, in the overall population and stratified by sex; Figure S9: Univariate exposure–response plots with the 95% credible intervals for the effect of each individual PFAS when others are fixed at their median in the BKMR model; Figure S10: Overall association of the mixture of five PFAS with the risk of psoriasis in the BKMR model. The figure plots the estimated difference in the risk of psoriasis when all PFAS concentrations are fixed at a particular percentile quantile (ranging from 0.25 to 0.75), as compared to when PFAS concentrations are fixed at the 50th percentile (reference); Figure S11: Association of each individual PFAS with the risk of psoriasis in the BKMR model. This figure describes the estimated difference in risk of psoriasis associated with a change in each individual PFAS from its 25th to 75th percentile when all the other PFAS are fixed at either the 25th 50th, or 75th percentile; Table S1: Distribution of serum per- and polyfluoroalkyl substance (PFAS) among participants, NHANES 2003–2018 (N = 5370); Table S2: Subgroup analysis of the association between PFOA exposure and risk of psoriasis; Table S3: Subgroup analysis of the association between PFOS exposure and risk of psoriasis; Table S4, Subgroup analysis of the association between PFHxS exposure and risk of psoriasis; Table S5: Subgroup analysis of the association between PFDA exposure and risk of psoriasis; Table S6: Subgroup analysis of the association between PFNA exposure and risk of psoriasis.
