# Supplementary material for: Exposure to Per- and Polyfluoroalkyl Substances and Risk of Psoriasis: A Population-Based Study
Source: Toxics. 2024 Nov 19;12(11):828. doi: 10.3390/toxics12110828 (PMC11598214; doi:10.3390/toxics12110828)

Supplemental Table S1. Distribution of serum per- and polyfluoroalkyl substance (PFAS) among participants, NHANES 2003–2018 (*N* = 5370).

**PFAS (ng/mL)**	**Detection frequency (%)**	**GM**	**Mean**	**Percentile**				
				**5th**	**25th**	**50th**	**75th**	**95th**
PFOA	99.53	2.88	3.65	0.77	1.77	2.70	4.27	7.70
PFOS	99.67	9.84	14.57	1.90	5.30	9.80	17.10	37.12
PFHxS	98.40	1.55	2.30	0.30	0.88	1.50	2.60	6.00
PFDA	79.95	0.25	0.36	0.07	0.19	0.20	0.40	0.95
PFNA	99.24	0.97	1.25	0.30	0.66	0.98	1.48	3.03

Abbreviations: GM, geometric mean; PFOA, perfluorooctanoic acid; PFOS, perfluorooctane sulfonate; PFHxS, perfluorohexane sulfonic acid; PFDA, perfluorodecanoic acid; PFNA, perfluorononanoic acid.

Supplemental Table S2. Subgroup analysis of the association between PFOA exposure and risk of psoriasis.

Characteristic	Continuous		T1	T2		T3		*p*-value
	OR (95% CI)	*p-value*		OR (95% CI)	*p-value*	OR (95% CI)	*p-value*	for trend
**Age**								
<60	1.16 (0.90, 1.50)	0.26	Ref	1.32 (0.68, 2.57)	0.41	1.69 (0.79, 3.64)	0.18	0.16
≥60	1.83 (1.29, 2.59)	**0.001**	Ref	2.82 (1.04, 7.69)	**0.04**	4.62 (1.78, 12.0)	**0.003**	**0.005**
**Sex**								
Male	1.03 (0.77, 1.37)	0.85	Ref	1.39 (0.56, 3.43)	0.47	1.28 (0.51, 3.24)	0.59	0.67
Female	1.52 (1.15, 2.00)	**0.004**	Ref	1.39 (0.66, 2.91)	0.38	3.12 (1.46, 6.64)	**0.004**	**0.004**
**Poverty income ratio**								
≥1.85	1.30 (1.00, 1.70)	**0.05**	Ref	1.47 (0.67, 3.25)	0.33	2.27 (0.96, 5.35)	0.06	**0.05**
<1.85	1.01 (0.68, 1.49)	0.98	Ref	1.45 (0.64, 3.32)	0.37	1.05 (0.40, 2.79)	0.92	0.85
**Marital status**								
Unmarried or other	1.09 (0.74, 1.59)	0.67	Ref	1.69 (0.72, 4.01)	0.23	1.78 (0.73, 4.30)	0.20	0.23
Married or living with a partner	1.30 (0.98, 1.72)	0.07	Ref	1.36 (0.60, 3.07)	0.46	2.02 (0.82, 4.94)	0.12	0.10
**Educational attainment**								
<High school	0.95 (0.63, 1.45)	0.81	Ref	0.70 (0.24, 2.03)	0.50	0.84 (0.28, 2.55)	0.75	0.74
≥High school	1.29 (1.00, 1.65)	**0.05**	Ref	1.74 (0.87, 3.51)	0.12	2.36 (1.10, 5.05)	**0.03**	**0.02**
**BMI**								
>24.9	1.13 (0.89, 1.44)	0.33	Ref	1.11 (0.58, 2.14)	0.75	1.57 (0.77, 3.20)	0.22	0.19
≤24.9	1.63 (1.09, 2.44)	**0.02**	Ref	4.43 (1.09, 18.0)	**0.04**	4.39 (0.95, 20.3)	0.06	0.07
**Smoking status**								
Never	1.45 (1.03, 2.03)	**0.03**	Ref	1.66 (0.71, 3.87)	0.24	2.92 (1.30, 6.55)	**0.01**	**0.01**
Former	0.95 (0.62, 1.44)	0.79	Ref	1.04 (0.37, 2.89)	0.95	1.00 (0.30, 3.33)	0.99	0.99
Current	1.35 (0.99, 1.85)	0.06	Ref	1.64 (0.51, 5.26)	0.40	2.44 (0.86, 6.92)	0.09	0.08

**Alcohol consumption**								
Never	1.38 (0.81, 2.32)	0.23	Ref	1.36 (0.21, 9.08)	0.75	1.65 (0.41, 6.66)	0.48	0.50
Former	1.48 (0.97, 2.28)	0.07	Ref	1.46 (0.39, 5.43)	0.57	3.20 (0.91, 11.2)	0.07	**0.05**
Mild	1.15 (0.70, 1.89)	0.57	Ref	1.12 (0.46, 2.69)	0.80	1.56 (0.54, 4.49)	0.40	0.38
Moderate	1.11 (0.74, 1.68)	0.61	Ref	0.97 (0.24, 3.81)	0.96	1.47 (0.42, 5.17)	0.55	0.51
Heavy	1.38 (0.88, 2.18)	0.16	Ref	3.03 (0.54, 17.0)	0.21	2.66 (0.40, 17.6)	0.31	0.30
**Physical activity**								
No	1.17 (0.85,1.60)	0.33	Ref	1.44 (0.65, 3.19)	0.37	1.64 (0.71, 3.78)	0.24	0.25
Yes	1.29 (0.95,1.74)	0.10	Ref	1.48 (0.57, 3.86)	0.41	2.26 (0.82, 6.28)	0.11	0.08

Supplemental Table S3. Subgroup analysis of the association between PFOS exposure and risk of psoriasis.

Characteristic	Continuous		T1	T2		T3		P value for trend
	OR (95% CI)	*p-value*		OR (95% CI)	*p-value*	OR (95% CI)	*p-value*	
**Age**								
<60	1.18 (0.99, 1.40)	0.07	Ref	1.33 (0.68, 2.61)	0.40	1.66 (0.92, 3.00)	0.09	0.09
≥60	1.29 (0.92, 1.80)	0.14	Ref	1.78 (0.36, 8.91)	0.47	2.53 (0.53, 12.1)	0.24	0.20
**Sex**								
Male	1.12 (0.91, 1.37)	0.28	Ref	1.32 (0.53, 3.30)	0.55	1.34 (0.58, 3.06)	0.49	0.54
Female	1.30 (0.99, 1.70)	0.06	Ref	1.28 (0.59, 2.80)	0.52	2.41 (1.06, 5.46)	**0.04**	**0.04**
**Poverty income ratio**								
≥1.85	1.31 (1.06, 1.61)	**0.01**	Ref	1.58 (0.66, 3.76)	0.30	2.40 (1.09, 5.29)	**0.03**	**0.03**
<1.85	0.92 (0.75, 1.14)	0.44	Ref	1.16 (0.48, 2.77)	0.74	0.54 (0.20, 1.51)	0.24	0.30
**Marital status**								
Unmarried or other	0.98 (0.79, 1.21)	0.82	Ref	1.36 (0.56, 3.30)	0.50	0.97 (0.40, 2.34)	0.94	0.95
Married or living with a partner	1.33 (1.08, 1.63)	**0.007**	Ref	1.43 (0.62, 3.30)	0.40	2.31 (1.13, 4.74)	**0.02**	**0.02**
**Educational attainment**								
<High school	1.16 (0.92, 1.46)	0.22	Ref	3.07 (1.15, 8.19)	**0.03**	1.51 (0.50, 4.57)	0.46	0.54
≥High school	1.20 (1.00, 1.45)	0.06	Ref	1.19 (0.58, 2.47)	0.63	1.76 (0.94, 3.29)	0.08	0.07
**BMI**								
>24.9	1.16 (0.97, 1.38)	0.10	Ref	1.27 (0.61, 2.64)	0.53	1.65 (0.90, 3.01)	0.10	0.09
≤24.9	1.20 (0.85, 1.71)	0.29	Ref	1.50 (0.51, 4.44)	0.45	1.62 (0.48, 5.49)	0.43	0.45
**Smoking status**								
Never	1.25 (1.00, 1.57)	0.06	Ref	1.33 (0.54, 3.28)	0.54	1.87 (0.80, 4.34)	0.14	0.13
Former	1.14 (0.80, 1.61)	0.47	Ref	1.10 (0.40, 2.99)	0.86	1.61 (0.54, 4.84)	0.39	0.35
Current	1.28 (0.96, 1.72)	0.10	Ref	2.04 (0.48, 8.74)	0.33	1.95 (0.66, 5.77)	0.22	0.21

**Alcohol consumption**								
Never	0.96 (0.61, 1.50)	0.85	Ref	0.72 (0.17, 3.01)	0.64	0.77 (0.16, 3.63)	0.73	0.80
Former	1.52 (1.11, 2.09)	**0.01**	Ref	1.15 (0.30, 4.40)	0.84	2.73 (1.36, 5.48)	**0.006**	**0.006**
Mild	1.29 (0.97, 1.71)	0.08	Ref	2.57 (0.75, 8.79)	0.13	3.19 (0.99, 10.3)	**0.05**	**0.05**
Moderate	1.12 (0.82, 1.54)	0.47	Ref	0.91 (0.29, 2.87)	0.87	1.32 (0.40, 4.32)	0.64	0.62
Heavy	0.96 (0.73, 1.26)	0.76	Ref	1.29 (0.41, 4.07)	0.66	0.88 (0.27, 2.87)	0.83	0.88
**Physical activity**								
No	1.24 (1.02,1.50)	0.03	Ref	1.82 (0.78, 4.22)	0.16	2.09 (1.01, 4.33)	**0.05**	**0.04**
Yes	1.17 (0.92,1.49)	0.21	Ref	1.07 (0.44, 2.58)	0.88	1.53 (0.68, 3.48)	0.30	0.28

Supplemental Table S4. Subgroup analysis of the association between PFHxS exposure and risk of psoriasis.

Characteristic	Continuous		T1	T2		T3		P value for trend
	OR (95% CI)	*p-value*		OR (95% CI)	*p-value*	OR (95% CI)	*p-value*	
**Age**								
<60	1.14 (0.93, 1.39)	0.21	Ref	1.36 (0.83, 2.23)	0.21	1.72 (0.99, 3.01)	0.06	0.07
≥60	1.09 (0.85, 1.41)	0.48	Ref	1.49 (0.43, 5.15)	0.52	1.95 (0.48, 7.92)	0.34	0.35
**Sex**								
Male	1.07 (0.81, 1.42)	0.63	Ref	1.45 (0.58, 3.60)	0.42	1.51 (0.59, 3.91)	0.39	0.45
Female	1.16 (0.91, 1.48)	0.22	Ref	1.22 (0.58, 2.55)	0.60	2.08 (0.98, 4.40)	0.06	0.06
**Poverty income ratio**								
≥1.85	1.25 (0.98, 1.60)	0.07	Ref	1.57 (0.80, 3.08)	0.19	2.26 (1.03, 4.95)	**0.04**	**0.04**
<1.85	0.84 (0.66, 1.07)	0.16	Ref	1.00 (0.42, 2.42)	0.99	0.66 (0.24, 1.76)	0.40	0.40
**Marital status**								
Unmarried or other	0.97 (0.79, 1.19)	0.75	Ref	1.22 (0.52, 2.90)	0.64	1.57 (0.70, 3.53)	0.27	0.29
Married or living with a partner	1.20 (0.95, 1.51)	0.13	Ref	1.42 (0.78, 2.59)	0.25	1.72 (0.88, 3.36)	0.11	0.13
**Educational attainment**								
<High school	0.95 (0.68, 1.32)	0.76	Ref	0.67 (0.25, 1.79)	0.42	0.95 (0.29, 3.17)	0.94	0.99
≥High school	1.15 (0.94, 1.40)	0.19	Ref	1.49 (0.87, 2.58)	0.15	1.84 (1.03, 3.28)	**0.04**	**0.05**
**BMI**								
>24.9	1.04 (0.83, 1.28)	0.75	Ref	1.17 (0.75, 1.82)	0.48	1.12 (0.63, 1.99)	0.70	0.76
≤24.9	1.32 (1.07, 1.64)	**0.01**	Ref	1.24 (0.25, 6.19)	0.79	5.00 (1.62, 15.4)	**0.006**	**0.005**
**Smoking status**								
Never	1.10 (0.88, 1.36)	0.41	Ref	1.95 (0.94, 4.04)	0.07	2.56 (1.15, 5.68)	**0.02**	**0.03**
Former	1.19 (0.79, 1.79)	0.40	Ref	1.65 (0.57, 4.75)	0.35	1.60 (0.55, 4.63)	0.38	0.46
Current	0.95 (0.60, 1.51)	0.82	Ref	0.27 (0.11, 0.65)	**0.004**	0.56 (0.16, 1.93)	0.35	0.47

**Alcohol consumption**								
Never	1.18 (0.77, 1.82)	0.43	Ref	1.42 (0.18, 11.4)	0.73	4.88 (0.89, 26.8)	0.07	**0.03**
Former	1.31 (1.01, 1.70)	**0.05**	Ref	0.76 (0.28, 2.06)	0.58	2.19 (0.98, 4.89)	0.06	0.06
Mild	0.95 (0.68, 1.32)	0.74	Ref	1.62 (0.61, 4.36)	0.33	1.17 (0.35, 3.88)	0.79	0.98
Moderate	1.34 (0.87, 2.05)	0.18	Ref	1.40 (0.41, 4.73)	0.59	2.70 (0.95, 7.70)	0.06	**0.03**
Heavy	0.98 (0.74, 1.28)	0.85	Ref	1.29 (0.38, 4.37)	0.68	0.70 (0.17, 2.89)	0.62	0.64
**Physical activity**								
No	1.02 (0.78,1.34)	0.89	Ref	1.36 (0.67, 2.77)	0.39	1.27 (0.50, 3.26)	0.61	0.65
Yes	1.19 (0.92,1.53)	0.19	Ref	1.31 (0.62, 2.74)	0.47	1.96 (1.01, 3.82)	**0.05**	**0.04**

Supplemental Table S5. Subgroup analysis of the association between PFDA exposure and risk of psoriasis.

Characteristic	Continuous		T1	T2		T3		P value for trend
	OR (95% CI)	*p-value*		OR (95% CI)	*p-value*	OR (95% CI)	*p-value*	
**Age**								
<60	1.00 (0.80, 1.24)	0.97	Ref	0.47 (0.26, 0.84)	**0.01**	0.80 (0.45, 1.44)	0.45	0.40
≥60	1.14 (0.73, 1.78)	0.55	Ref	0.68 (0.22, 2.09)	0.49	1.21 (0.33, 4.47)	0.77	0.72
**Sex**								
Male	0.93 (0.71, 1.22)	0.60	Ref	0.29 (0.14, 0.57)	**<0.001**	0.57 (0.28, 1.14)	0.11	0.12
Female	1.17 (0.93, 1.47)	0.18	Ref	0.85 (0.39, 1.86)	0.68	1.48 (0.77, 2.85)	0.24	0.29
**Poverty income ratio**								
≥1.85	1.05 (0.84, 1.32)	0.67	Ref	0.45 (0.23, 0.87)	**0.02**	0.93 (0.51, 1.69)	0.80	0.85
<1.85	0.97 (0.71, 1.31)	0.83	Ref	0.67 (0.35, 1.26)	0.21	0.53 (0.20, 1.42)	0.20	0.14
**Marital status**								
Unmarried or other	1.05 (0.78, 1.41)	0.76	Ref	0.73 (0.33, 1.64)	0.44	1.21 (0.52, 2.79)	0.66	0.71
Married or living with a partner	1.03 (0.81, 1.31)	0.80	Ref	0.42 (0.21, 0.83)	**0.01**	0.77 (0.42, 1.42)	0.40	0.37
**Educational attainment**								
<High school	1.11 (0.89, 1.39)	0.34	Ref	1.31 (0.53, 3.24)	0.56	1.15 (0.48, 2.77)	0.76	0.73
≥High school	1.01 (0.81, 1.25)	0.95	Ref	0.43 (0.23, 0.78)	**0.006**	0.81 (0.47, 1.42)	0.46	0.44
**BMI**								
>24.9	1.00 (0.82, 1.24)	0.97	Ref	0.53 (0.30, 0.92)	**0.03**	0.83 (0.46, 1.47)	0.51	0.48
≤24.9	0.97 (0.61, 1.56)	0.91	Ref	0.27 (0.10, 0.74)	0.01	0.73 (0.21, 2.57)	0.62	0.64
**Smoking status**								
Never	1.04 (0.82, 1.32)	0.75	Ref	0.34 (0.16, 0.72)	**0.005**	0.77 (0.41, 1.44)	0.40	0.40
Former	0.90 (0.62, 1.31)	0.57	Ref	0.34 (0.14, 0.82)	**0.02**	0.62 (0.27, 1.43)	0.26	0.28
Current	1.39 (1.11, 1.75)	**0.005**	Ref	1.90 (0.53, 6.81)	0.32	2.49 (0.95, 6.52)	0.06	**0.05**

**Alcohol consumption**								
Never	0.84 (0.39, 1.84)	0.66	Ref	0.22 (0.03, 1.99)	0.18	0.43 (0.09, 2.11)	0.29	0.27
Former	1.09 (0.69, 1.72)	0.70	Ref	0.33 (0.11, 1.00)	**0.05**	0.99 (0.30, 3.27)	0.98	0.87
Mild	1.02 (0.73, 1.43)	0.89	Ref	0.46 (0.20, 1.03)	0.06	0.75 (0.32, 1.74)	0.50	0.56
Moderate	0.87 (0.60, 1.25)	0.44	Ref	0.42 (0.13, 1.38)	0.15	0.81 (0.25, 2.57)	0.71	0.74
Heavy	1.19 (0.78, 1.83)	0.41	Ref	0.72 (0.20, 2.61)	0.61	1.13 (0.31, 4.08)	0.85	0.90
**Physical activity**								
No	1.18 (0.95, 1.46)	0.13	Ref	1.05 (0.53, 2.08)	0.90	1.16 (0.55, 2.43)	0.69	0.70
Yes	0.90 (0.68, 1.20)	0.46	Ref	0.25 (0.11, 0.54)	**<0.001**	0.69 (0.35, 1.34)	0.26	0.28

Supplemental Table S6. Subgroup analysis of the association between PFNA exposure and risk of psoriasis.

Characteristic	Continuous		T1	T2		T3		P value for trend
	OR (95% CI)	*p-value*		OR (95% CI)	*p-value*	OR (95% CI)	*p-value*	
**Age**								
<60	1.10 (0.89, 1.36)	0.39	Ref	0.87 (0.50, 1.54)	0.63	1.36 (0.76, 2.42)	0.29	0.29
≥60	1.34 (0.94, 1.90)	0.10	Ref	0.98 (0.31, 3.14)	0.97	1.34 (0.36, 4.96)	0.65	0.63
**Sex**								
Male	0.98 (0.73, 1.30)	0.87	Ref	0.72 (0.34, 1.54)	0.39	0.93 (0.43, 1.99)	0.85	0.93
Female	1.33 (1.08, 1.65)	**0.01**	Ref	0.97 (0.46, 2.05)	0.94	1.98 (1.18, 3.30)	**0.01**	**0.02**
**Poverty income ratio**								
≥1.85	1.21 (0.99, 1.49)	0.07	Ref	0.90 (0.48, 1.71)	0.74	1.39 (0.77, 2.52)	0.27	0.24
<1.85	0.94 (0.62, 1.40)	0.75	Ref	0.81 (0.37, 1.75)	0.58	1.15 (0.50, 2.66)	0.74	0.83
**Marital status**								
Unmarried or other	0.99 (0.72, 1.37)	0.97	Ref	0.95 (0.40, 2.26)	0.91	1.04 (0.46, 2.34)	0.93	0.94
Married or living with a partner	1.20 (0.95, 1.51)	0.13	Ref	0.88 (0.44, 1.77)	0.72	1.47 (0.77, 2.83)	0.24	0.22
**Educational attainment**								
<High school	1.03 (0.77, 1.38)	0.84	Ref	1.79 (0.58, 5.47)	0.30	1.11 (0.42, 2.91)	0.83	0.79
≥High school	1.16 (0.94, 1.44)	0.17	Ref	0.77 (0.42, 1.38)	0.37	1.35 (0.78, 2.35)	0.28	0.26
**BMI**								
>24.9	1.15 (0.93, 1.41)	0.19	Ref	0.70 (0.38, 1.30)	0.26	1.36 (0.75, 2.47)	0.31	0.27
≤24.9	0.97 (0.66, 1.41)	0.85	Ref	1.42 (0.51, 3.93)	0.49	0.73 (0.21, 2.52)	0.61	0.64
**Smoking status**								
Never	1.15 (0.91, 1.45)	0.25	Ref	0.78 (0.37, 1.64)	0.51	1.26 (0.66, 2.40)	0.47	0.46
Former	1.09 (0.75, 1.58)	0.65	Ref	0.80 (0.30, 2.14)	0.65	0.97 (0.35, 2.70)	0.96	0.99
Current	1.21 (0.75, 1.96)	0.43	Ref	1.57 (0.43, 5.77)	0.49	3.04 (1.16, 7.95)	**0.02**	**0.02**

**Alcohol consumption**								
Never	1.25 (0.88, 1.76)	0.21	Ref	2.78 (0.58, 13.4)	0.20	1.55 (0.42, 5.67)	0.50	0.50
Former	1.03 (0.77, 1.38)	0.85	Ref	0.43 (0.12, 1.49)	0.18	1.40 (0.50, 3.95)	0.52	0.53
Mild	1.27 (0.90, 1.79)	0.18	Ref	0.84 (0.38, 1.89)	0.67	1.19 (0.49, 2.90)	0.69	0.64
Moderate	0.84 (0.56, 1.26)	0.40	Ref	0.79 (0.23, 2.73)	0.70	0.90 (0.30, 2.73)	0.86	0.87
Heavy	1.26 (0.85, 1.87)	0.24	Ref	1.09 (0.30, 3.99)	0.90	2.23 (0.60, 8.23)	0.23	0.23
**Physical activity**								
No	1.05 (0.78, 1.40)	0.76	Ref	1.17 (0.52, 2.62)	0.70	1.43 (0.71, 2.91)	0.31	0.32
Yes	1.22 (0.91, 1.63)	0.18	Ref	0.70 (0.33, 1.49)	0.35	1.29 (0.59, 2.81)	0.52	0.48

Supplemental Fig. S1. Flowchart of participants selection from the NHANES 2003–2018.

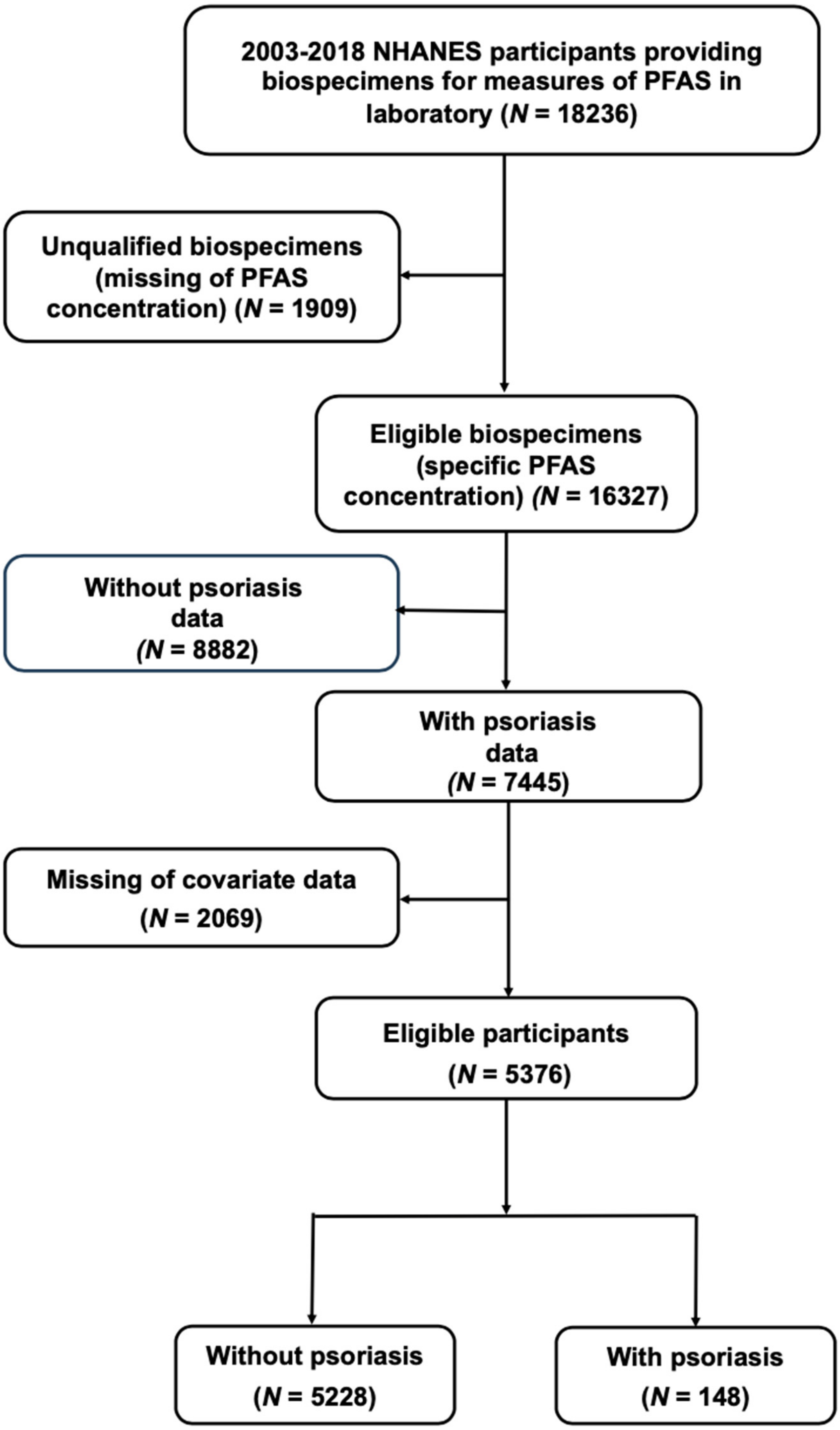

Supplemental Fig. S2. Directed Acyclic Graph (DAG) for covariate selection.

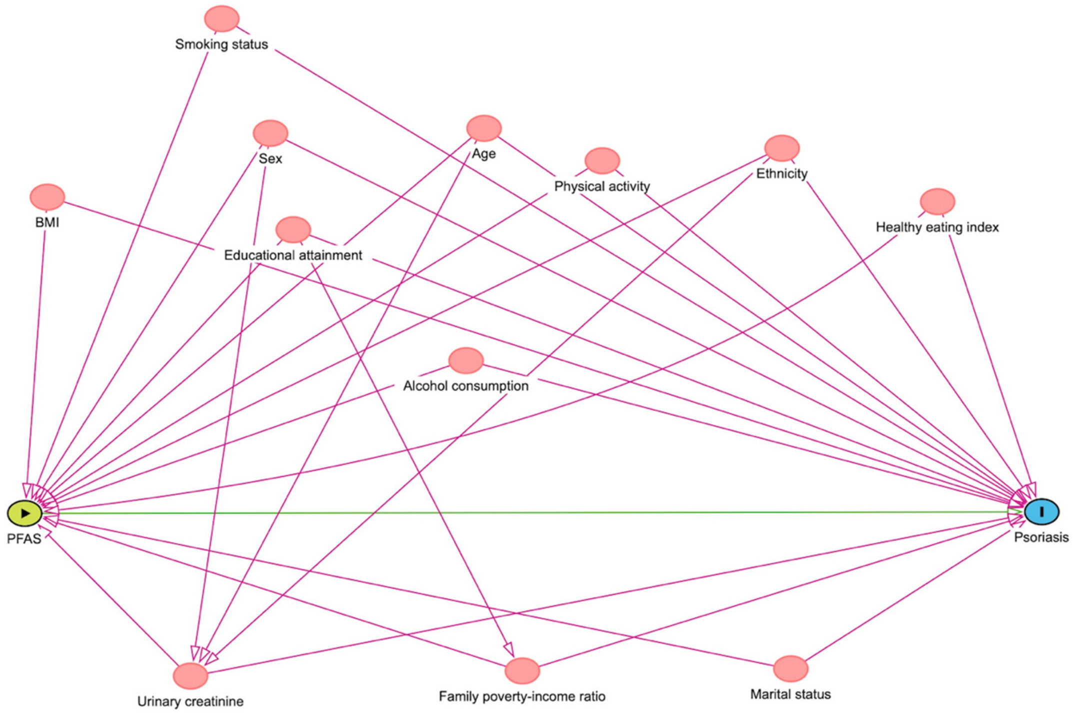

Supplemental Fig. S3. The restricted cubic spline analysis of dose-response relationships between PFOA and risk of psoriasis for (A) Male and (B) Female. The horizontal dotted lines represent the OR = 1. Solid lines indicate ORs, and shadow shape indicate 95% CIs. The 5th of PFOA was assigned as the reference point, with knots set at the 5th, 35th, 65th, and 95th percentiles of the log_2 transformed PFOA concentration. All models were adjusted for age, sex, race/ethnicity, marital status, PIR, educational attainment, smoking status, alcohol consumption, HEI, BMI, physical activity, and urinary creatinine. Abbreviations: PFOA: perfluorooctanoic acid; OR, odds ratio; CI, confidence interval; PIR, poverty income ratio; HEI, healthy eating index; BMI, body mass index.

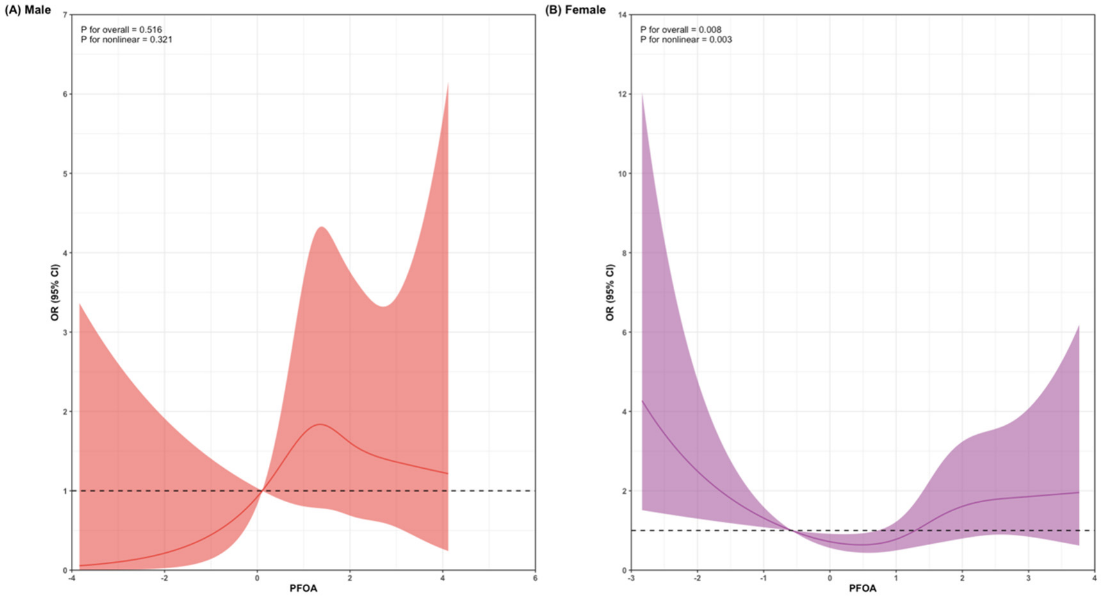

Supplemental Fig. S4. The restricted cubic spline analysis of dose-response relationships between PFOS and risk of psoriasis for (A) Male and (B) Female. The horizontal dotted lines represent the OR = 1. Solid lines indicate ORs, and shadow shape indicate 95% CIs. The 5th of PFOS was assigned as the reference point, with knots set at the 5th, 35th, 65th, and 95th percentiles of the log_2 transformed PFOS concentration. All models were adjusted for age, sex, race/ethnicity, marital status, PIR, educational attainment, smoking status, alcohol consumption, HEI, BMI, physical activity, and urinary creatinine. Abbreviations: PFOS: perfluorooctane sulfonic acid; OR, odds ratio; CI, confidence interval; PIR, poverty income ratio; HEI, healthy eating index; BMI, body mass index.

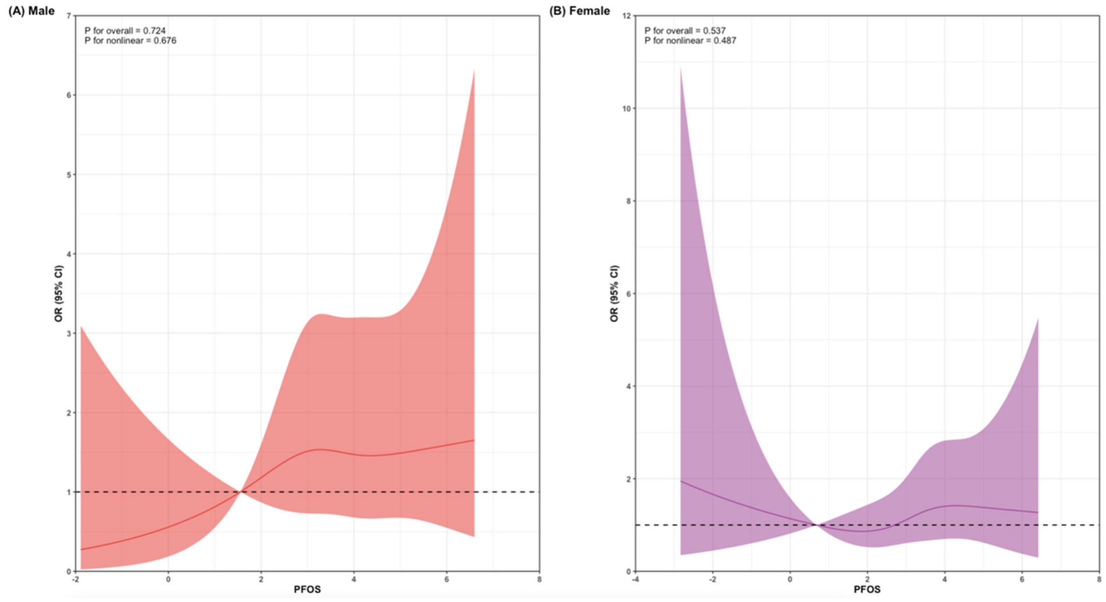

Supplemental Fig. S5. The restricted cubic spline analysis of dose-response relationships between PFHxS and risk of psoriasis for (A) Male and (B) Female. The horizontal dotted lines represent the OR = 1. Solid lines indicate ORs, and shadow shape indicate 95% CIs. The 5th of PFHxS was assigned as the reference point, with knots set at the 5th, 35th, 65th, and 95th percentiles of the log_2 transformed PFHxS concentration. All models were adjusted for age, sex, race/ethnicity, marital status, PIR, educational attainment, smoking status, alcohol consumption, HEI, BMI, physical activity, and urinary creatinine. Abbreviations: PFHxS: perfluorohexane sulfonate; OR, odds ratio; CI, confidence interval; PIR, poverty income ratio; HEI, healthy eating index; BMI, body mass index.

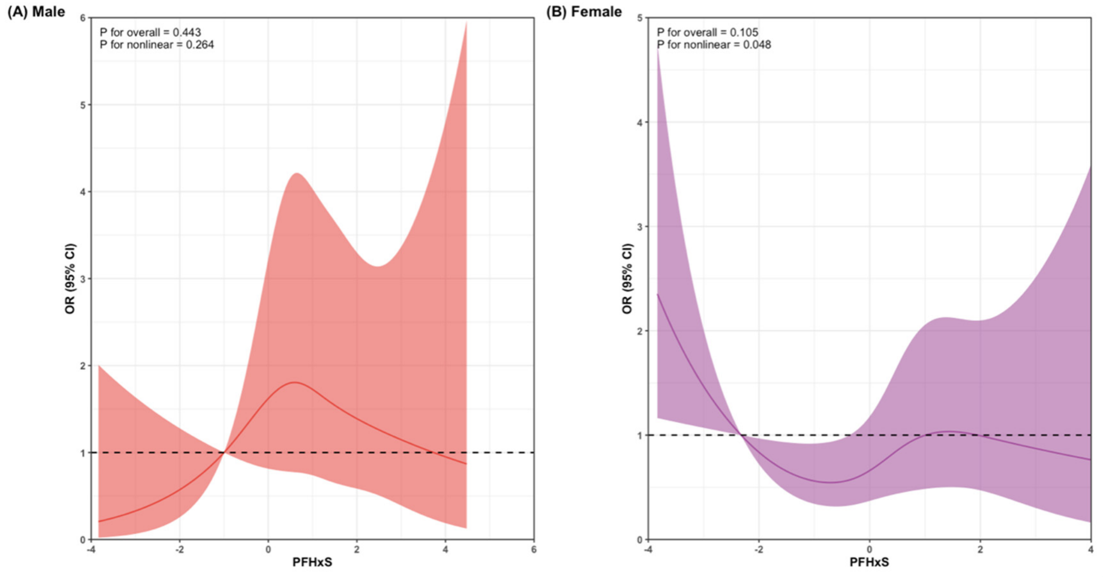

Supplemental Fig. S6. The restricted cubic spline analysis of dose-response relationships between PFDA and risk of psoriasis for (A) Male and (B) Female. The horizontal dotted lines represent the OR = 1. Solid lines indicate ORs, and shadow shape indicate 95% CIs. The 5th of PFDA was assigned as the reference point, with knots set at the 5th, 35th, 65th, and 95th percentiles of the $\log_2$ transformed PFDA concentration. All models were adjusted for age, sex, race/ethnicity, marital status, PIR, educational attainment, smoking status, alcohol consumption, HEI, BMI, physical activity, and urinary creatinine. Abbreviations: PFDA: perfluorodecanoic acid; OR, odds ratio; CI, confidence interval; PIR, poverty income ratio; HEI, healthy eating index; BMI, body mass index.

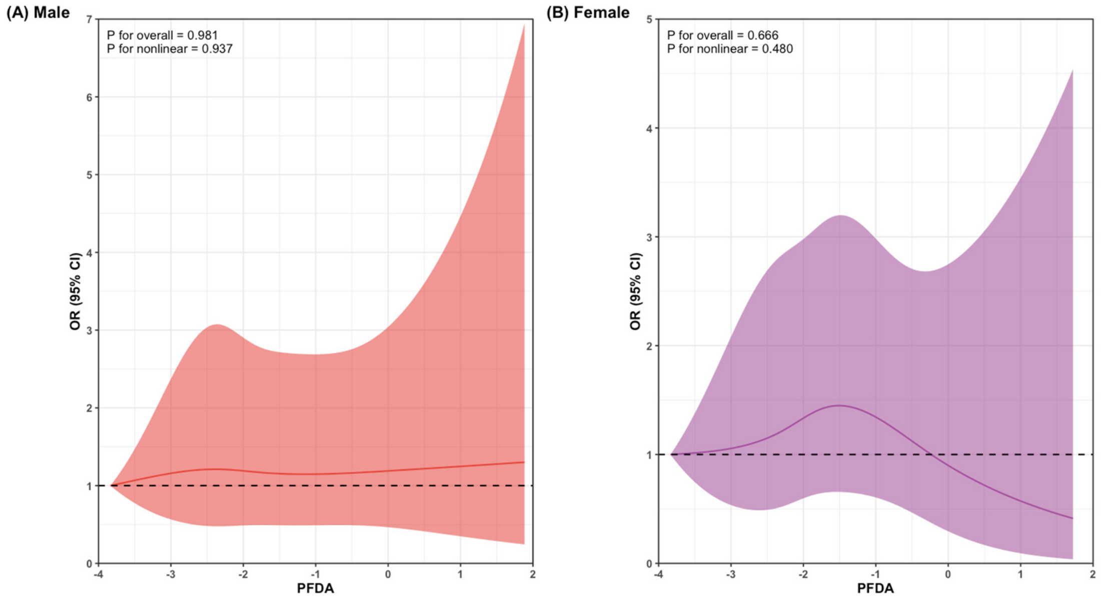

Supplemental Fig. S7. The restricted cubic spline analysis of dose-response relationships between PFNA and risk of psoriasis for (A) Male and (B) Female. The horizontal dotted lines represent the OR = 1. Solid lines indicate ORs, and shadow shape indicate 95% CIs. The 5th of PFNA was assigned as the reference point, with knots set at the 5th, 35th, 65th, and 95th percentiles of the log_2 transformed PFNA concentration. All models were adjusted for age, sex, race/ethnicity, marital status, PIR, educational attainment, smoking status, alcohol consumption, HEI, BMI, physical activity, and urinary creatinine. Abbreviations: PFNA: perfluorononanoic acid; OR, odds ratio; CI, confidence interval; PIR, poverty income ratio; HEI, healthy eating index; BMI, body mass index.

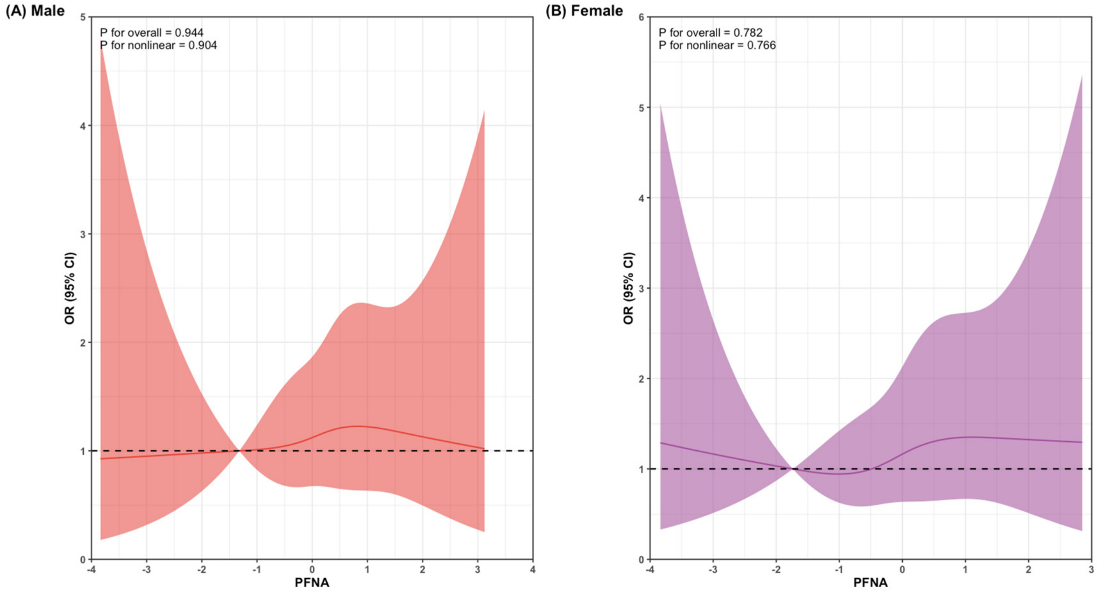

Supplemental Fig. S8. Positive and negative weights representing the partial effects of individual PFAS in the mixture on psoriasis risk, estimated using quantile g-computation, in the overall population and stratified by sex.

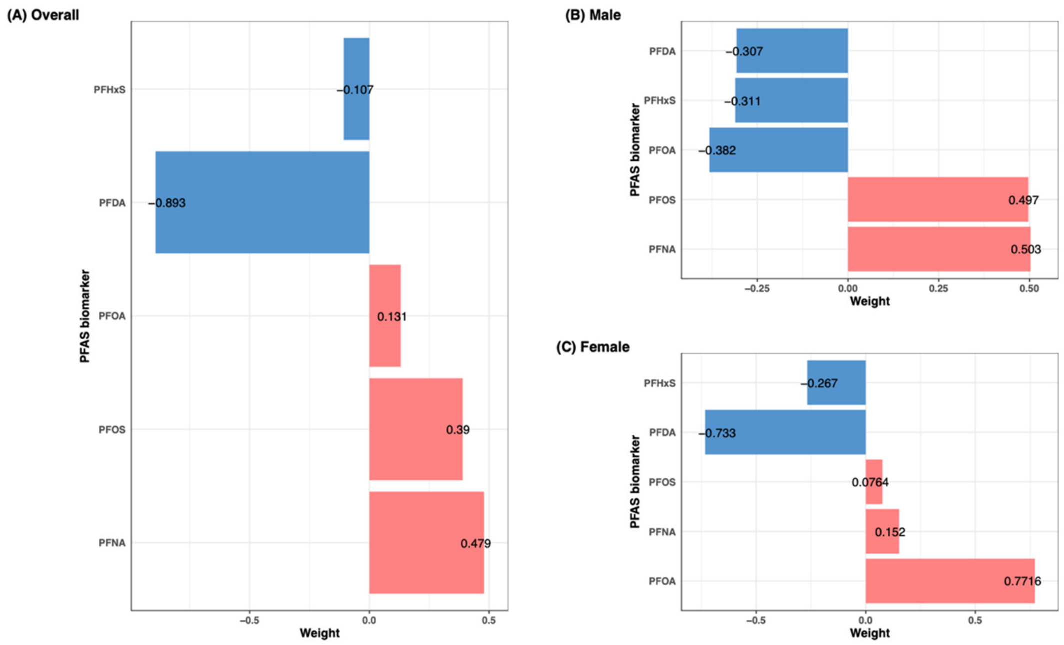

Supplemental Fig. S9. Univariate exposure–response plots with the 95% credible intervals for the effect of each individual PFAS when others are fixed at their median in BKMR model.

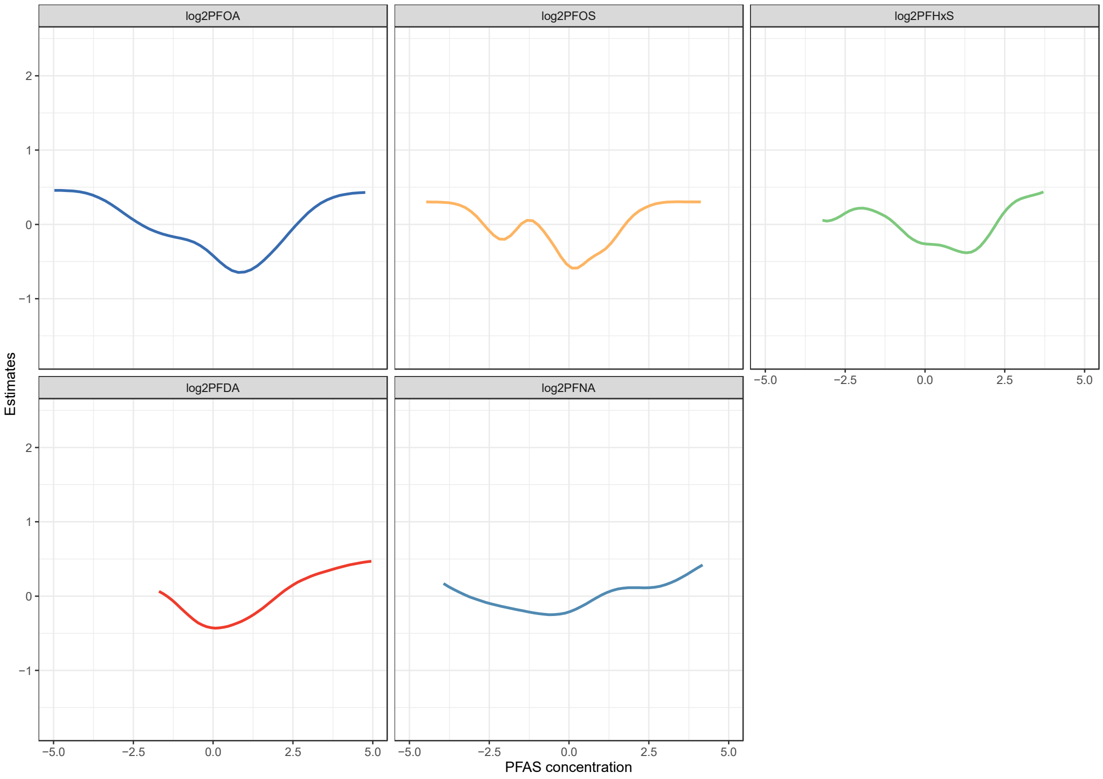

Supplemental Fig. S10. Overall association of the mixture of five PFAS with risk of psoriasis in BKMR model. The figure plots the estimated difference in risk of psoriasis when all PFAS concentrations are fixed at a particular percentile quantile (ranging from 0.25 to 0.75), as compared to when PFAS concentrations are fixed at the 50th percentile (reference). Dots indicate the estimate, and vertical lines indicate the 95% credible intervals.

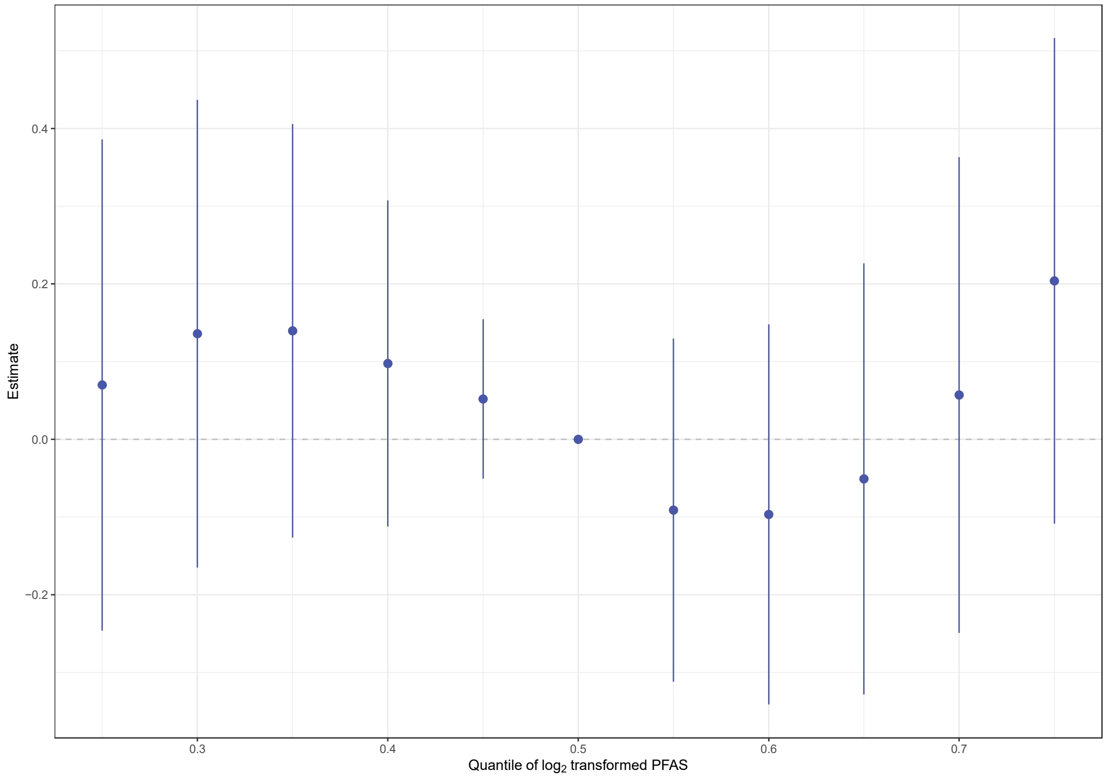

Supplemental Fig. S11. Associations of each individual PFAS with risk of psoriasis in BKMR model. This figure describes the estimated difference in risk of psoriasis associated with a change in each individual PFAS from its 25th to 75th percentile, when all the other PFAS are fixed at either the 25th 50th or 75th percentile. Dots indicate the estimate, and horizontal lines indicate the 95% credible intervals.

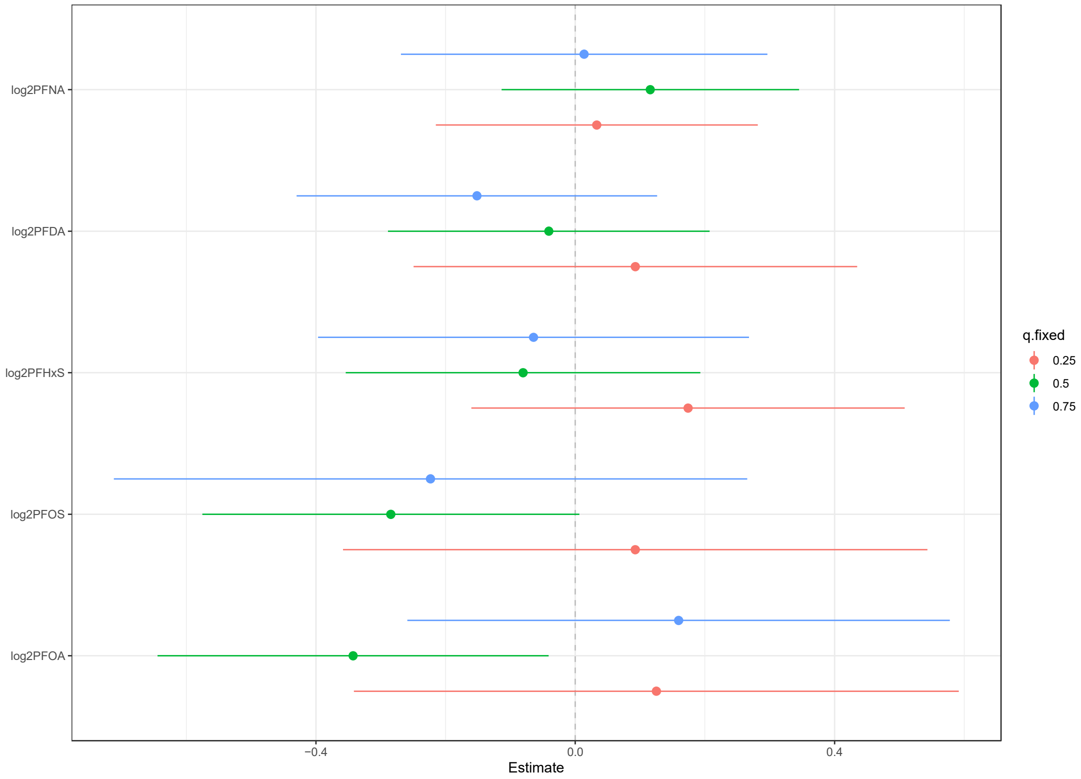

Supplement: Supplementary file 1 [file toxics-12-00828-s001.zip › toxics-3180915-supplementary.pdf]
